# Climate change and HIV prevention: Towards sustainable solutions – a narrative review

**DOI:** 10.1097/MD.0000000000042198

**Published:** 2025-04-18

**Authors:** Emmanuel Ifeanyi Obeagu, Isaac Isiko, Getrude Uzoma Obeagu

**Affiliations:** a Department of Biomedical and Laboratory Science, Africa University, Zimbabwe; b Department of Community Medicine, School of Public Health, NIMS University, Jaipur, Rajasthan, India; c School of Nursing Science, Kampala International University, Ishaka, Uganda.

**Keywords:** climate change, HIV prevention, public health, sustainable solutions, vulnerability

## Abstract

Climate change, with its pervasive environmental, social, and economic impacts, is emerging as a significant factor influencing global health outcomes, including the prevention and management of HIV. The intersection of these 2 critical issues presents unique challenges, particularly in terms of increased vulnerability among populations, disruptions to healthcare infrastructure, and the exacerbation of social inequalities. This narrative review examines the multifaceted relationship between climate change and HIV prevention, emphasizing the need for sustainable solutions that integrate climate resilience into public health strategies. The review highlights how climate change exacerbates vulnerabilities that contribute to the spread and impact of HIV. Factors such as displacement and migration due to extreme weather events, food insecurity from altered climate patterns, and economic instability directly affect individuals’ susceptibility to HIV and their access to necessary healthcare services. Additionally, the strain on healthcare infrastructure, resulting from climate-related damages and resource reallocations, further hinders effective HIV prevention and treatment efforts.

## 1. Introduction

Climate change is unequivocally one of the most pressing global challenges of our time, with profound implications for environmental stability, economic prosperity, and human health.^[1]^ The escalating frequency and intensity of extreme weather events, rising global temperatures, and sea-level rise are reshaping our planet and impacting all aspects of society. Among the myriads of health issues influenced by climate change, the prevention and management of HIV stand out as particularly vulnerable to these environmental shifts. This intersection of climate change and HIV prevention presents complex challenges that require integrated and sustainable solutions.^[2]^ HIV remains a significant global health crisis, affecting millions of people worldwide. Despite advancements in medical treatments and preventive measures, the disease continues to spread, particularly in regions already burdened by socio-economic challenges and healthcare disparities. The intersection of HIV and climate change creates a perfect storm of vulnerability, where the impacts of 1 exacerbate the other, leading to compounded health risks for already marginalized populations.^[3]^ Extreme weather events, such as hurricanes, floods, and droughts, displace large populations, disrupting their access to healthcare and continuity of care. Migrant populations often face barriers in accessing HIV prevention and treatment services, increasing their risk of infection. Moreover, changes in climate patterns affect agricultural productivity, leading to food insecurity and malnutrition.^[3]^ Malnutrition weakens the immune system, making individuals more susceptible to infections, including HIV. Economic instability, driven by climate change, further exacerbates the challenges of HIV prevention. Climate-induced economic disruptions, such as loss of livelihoods and increased poverty, limit financial resources for healthcare, including HIV services. In regions heavily dependent on agriculture or tourism, extreme weather events can devastate local economies, reducing the capacity of communities to fund and maintain health programs. This economic strain makes it harder for individuals to access and adhere to HIV prevention and treatment, increasing the risk of transmission and poor health outcomes.^[4]^

Healthcare infrastructure is also significantly impacted by climate change, posing direct challenges to HIV prevention and management. Extreme weather events can damage healthcare facilities, disrupt supply chains for medications, and hinder the delivery of essential health services. The reallocation of resources to address immediate climate-related emergencies often comes at the expense of long-term health programs, including those targeting HIV. Furthermore, the stress and burnout experienced by healthcare workers during climate crises can reduce the overall capacity of the healthcare system to respond effectively to HIV. To address these challenges, integrating climate resilience into HIV prevention strategies is essential.^[2]^ Strengthening healthcare systems to withstand climate impacts ensures that services remain accessible and functional during and after extreme weather events. This includes investing in robust infrastructure, developing emergency preparedness plans, and ensuring that supply chains for medications and other critical resources are resilient to disruptions.^[1,4,5]^ By building a resilient healthcare system, we can better protect vulnerable populations and maintain continuity of care. Community-based approaches play a crucial role in enhancing resilience and addressing the compounded risks of climate change and HIV. Empowering communities with resources, education, and support to adapt to climate changes can significantly improve local capacity for HIV prevention and care. Community-driven initiatives can tailor responses to specific local needs, ensuring that interventions are culturally appropriate and effective. Additionally, engaging community members in the planning and implementation of these initiatives fosters a sense of ownership and commitment, which is vital for the sustainability of these efforts.^[5]^

Policy and advocacy are also critical components in the intersection of climate change and HIV prevention. Developing policies that integrate climate resilience into health programs and advocating for sustainable funding can support long-term solutions. Policymakers must recognize the interconnectedness of these issues and allocate resources accordingly. International cooperation and global advocacy are necessary to mobilize the resources and political will needed to implement comprehensive strategies that address both climate change and HIV. Innovative approaches to addressing these dual challenges include leveraging technology and integrating programs. Mobile health technologies (mHealth)^[3]^ can deliver HIV services remotely, ensuring continuity of care during climate disruptions. Green healthcare initiatives, which implement environmentally sustainable practices, can reduce the carbon footprint of healthcare facilities while enhancing their resilience. Integrating HIV prevention into broader climate adaptation programs, such as incorporating HIV education into disaster preparedness efforts, can create synergies that benefit both health and environmental outcomes.^[6]^

## 2. Aim

The aim of this review is to explore the intricate relationship between climate change and HIV prevention, identifying the impacts of environmental changes on health systems and vulnerable populations.

## 3. Justification

The justification for exploring the intersection of climate change and HIV prevention stems from the urgent need to address the complexities posed by an increasingly interconnected world. Climate change disproportionately affects marginalized and vulnerable populations, who are already at higher risk for HIV infection. Disruptions caused by extreme weather events, food insecurity, and health system weaknesses can exacerbate vulnerabilities and hinder access to essential health services. Extreme weather events and climate-related disasters can disrupt healthcare delivery, including HIV prevention and treatment services. Research has shown that such disruptions can lead to increased treatment interruptions and poorer health outcomes for individuals living with HIV.^[7]^ By investigating the impacts of climate change on healthcare infrastructure, this review aims to identify strategies to enhance resilience and ensure the continuity of HIV services. The complex interplay between climate change and health necessitates integrated solutions that consider both environmental and health factors. Current HIV prevention strategies may not adequately account for climate-related challenges, leading to gaps in service delivery and increased risk for vulnerable populations. This review emphasizes the need for interdisciplinary approaches that integrate climate resilience into HIV prevention efforts, promoting more comprehensive and effective strategies.^[8]^ As policymakers increasingly recognize the impact of climate change on public health, there is a growing need for evidence-based policies that address these interconnected challenges. This review aims to provide practical recommendations for policymakers and healthcare providers, fostering a more supportive environment for climate-resilient HIV prevention strategies. By advocating for inclusive policies that address social determinants of health, this review seeks to promote health equity and improve health outcomes for affected communities.^[9]^ Community engagement is critical for developing effective HIV prevention strategies. Empowering local communities to address both climate change and health issues can lead to more sustainable and culturally appropriate solutions. This review underscores the importance of community-driven initiatives and capacity-building efforts in fostering resilience against climate change and enhancing HIV prevention outcomes.

## 4. Review methodology

### 4.1. Search strategy

A systematic search was conducted in academic databases and reputable sources, including:

- **Databases**: PubMed, Scopus, Web of Science, Google Scholar.
- **Keywords**: “climate change,” “HIV prevention,” “sustainability,” “health equity,” “vulnerable populations,” “healthcare infrastructure,” “climate resilience,” “sustainable development goals.”
- **Search Terms**: Boolean operators (e.g., AND, OR) were used to combine terms:
- “Climate change AND HIV prevention.”
- “Sustainable healthcare AND HIV.”
- “Extreme weather events AND healthcare access.”

## 5. Inclusion and exclusion criteria

- **Inclusion Criteria**:
- Articles published in English.
- Studies focusing on climate change’s impact on health systems, vulnerable populations, and HIV prevention strategies.
- **Exclusion Criteria**:
- Non-English publications.
- Studies without relevance to climate change or HIV prevention.
- Articles with insufficient methodological rigor.

## 6. Critical appraisal

Each study was appraised for quality and relevance using standardized tools, such as:

- The Newcastle-Ottawa Scale (NOS) for observational studies.
- PRISMA guidelines to ensure methodological transparency.

## 7. Limitations

- Limited data on the specific impacts of climate change on HIV prevention, especially in low- and middle-income countries.
- Variability in study methodologies may affect the generalizability of findings.

## 8. Climate Change and Increased Vulnerability to HIV

Climate change exacerbates vulnerabilities to HIV through a variety of interconnected pathways, significantly impacting populations already at risk. The consequences of climate change – such as displacement and migration, food insecurity, and economic instability – create environments where the spread and impact of HIV can intensify. Several studies have documented how climate change exacerbates vulnerability to HIV. For instance, a systematic review by Lestari et al found that climate-related factors, such as extreme weather events and changing ecosystems, contribute to social determinants of health that increase susceptibility to HIV infection. Communities affected by displacement due to climate change often experience disruptions in healthcare access and increased engagement in high-risk behaviors.^[10]^ Furthermore, climate change can exacerbate poverty and food insecurity, both of which are linked to higher rates of HIV transmission and lower health outcomes among affected populations.^[11]^

## 9. Displacement and migration

One of the most direct ways climate change increases vulnerabilities to HIV is through displacement and migration. Extreme weather events, such as hurricanes, floods, droughts, and rising sea levels, force large populations to relocate. Displaced individuals often move to temporary shelters or urban areas with limited resources, where overcrowding and poor living conditions prevail. These environments can disrupt access to healthcare services, including HIV prevention, testing, and treatment. Migrants and displaced populations face significant barriers to healthcare access.^[7]^ They may lack legal documentation, face language barriers, or encounter discrimination, making it difficult to obtain necessary health services. Displacement often leads to a breakdown in social networks and support systems, which are critical for maintaining health and accessing care. As a result, individuals in these situations are at increased risk of HIV infection and may struggle to adhere to treatment regimens if already living with HIV.^[4,7,8]^

## 10. Food insecurity

Climate change also contributes to food insecurity, which can directly and indirectly increase vulnerability to HIV. Changes in climate patterns, such as altered rainfall and temperature extremes, affect agricultural productivity, leading to crop failures and reduced food availability. Food insecurity and malnutrition weaken the immune system, making individuals more susceptible to infections, including HIV.^[8–10]^Malnutrition can also impact the effectiveness of antiretroviral therapy (ART) for those already living with HIV, as proper nutrition is essential for the body’s ability to process and benefit from medication. Additionally, food insecurity can force individuals, particularly women and girls, into transactional sex or other high-risk behaviors to obtain food, further increasing their risk of HIV infection. Addressing food insecurity as part of HIV prevention efforts is essential for reducing these compounded vulnerabilities.^[12,13]^

## 11. Economic instability

Economic instability driven by climate change further exacerbates the challenges of HIV prevention. Climate-induced economic disruptions, such as loss of livelihoods in agriculture, fisheries, and tourism, reduce financial resources for both individuals and healthcare systems. Poverty and economic hardship limit access to healthcare services, including HIV prevention, testing, and treatment.^[1,5]^In regions heavily dependent on climate-sensitive industries, economic instability can undermine the capacity of communities to fund and maintain health programs. This economic strain makes it harder for individuals to afford transportation to healthcare facilities, purchase necessary medications, or maintain consistent engagement with healthcare services. Economic instability also exacerbates social inequalities, disproportionately affecting marginalized groups who are already at higher risk of HIV.^[14,15]^

## 12. Social and behavioral factors

Climate change can influence social and behavioral factors that increase vulnerability to HIV. For example, the stress and trauma associated with extreme weather events and displacement can lead to increased substance use, mental health issues, and risky sexual behaviors.^[12]^ These factors can contribute to higher rates of HIV transmission. Furthermore, in times of crisis, traditional social structures and norms may break down, leading to increased gender-based violence and exploitation. Women and girls, in particular, may face higher risks of sexual violence and coercion, increasing their vulnerability to HIV. Addressing these social and behavioral factors is critical for comprehensive HIV prevention in the context of climate change.^[16,17]^

## 13. Impact on Healthcare Infrastructure

Climate change poses significant challenges to healthcare infrastructure, which is crucial for effective HIV prevention. Extreme weather events can damage healthcare facilities, disrupt supply chains for medications, and hinder the delivery of essential health services. Floods, hurricanes, and other natural disasters can lead to the destruction of clinics, hospitals, and medical supplies, making it difficult for affected populations to access care.^[3,9,14]^The reallocation of resources to address immediate climate-related emergencies often comes at the expense of long-term health programs, including those targeting HIV. During a crisis, funding and attention may be diverted from HIV prevention and treatment efforts to emergency response activities. This diversion can result in reduced availability of ART, interruptions in treatment, and increased risk of HIV transmission. The resilience of healthcare infrastructure is crucial for maintaining effective HIV prevention and treatment services in the face of climate change. Empirical evidence indicates that extreme weather events, such as floods and hurricanes, can significantly disrupt healthcare delivery. highlights how Hurricane Katrina impacted HIV care services in New Orleans, leading to increased treatment interruptions and adverse health outcomes for individuals living with HIV.^[18]^ This underscores the need for investments in climate-resilient healthcare facilities and systems to ensure continuity of care.^[19–21]^

## 14. Addressing compounded risks

To address the compounded risks of climate change and HIV, it is essential to integrate climate resilience into HIV prevention strategies. Strengthening healthcare systems to withstand climate impacts ensures that services remain accessible and functional during and after extreme weather events.^[22]^ Investing in resilient infrastructure, developing emergency preparedness plans, and ensuring robust supply chains for medications are crucial steps. Community-based approaches are vital for enhancing resilience and addressing the specific needs of vulnerable populations. Empowering communities with resources, education, and support to adapt to climate changes can improve local capacity for HIV prevention and care. Engaging community members in the planning and implementation of interventions ensures that responses are culturally appropriate and effective. Policy and advocacy are also critical components of addressing the intersection of climate change and HIV.^[23,24]^ Developing policies that integrate climate resilience into health programs and advocating for sustainable funding can support long-term solutions.^[17]^ Policymakers must recognize the interconnectedness of these issues and allocate resources accordingly. International cooperation and global advocacy are necessary to mobilize the resources and political will needed to implement comprehensive strategies.^[25]^

## 15. Impact on healthcare infrastructure

Climate change poses significant challenges to healthcare infrastructure, crucial for effective HIV prevention and management. The increasing frequency and severity of extreme weather events, such as hurricanes, floods, droughts, and wildfires, can cause extensive damage to healthcare facilities, disrupt medication supply chains, and hinder the delivery of essential health services. These disruptions have far-reaching consequences for HIV prevention, testing, and treatment, as well as for the overall resilience of healthcare systems in affected regions.^[26]^

## 16. Damage to healthcare facilities

Extreme weather events can physically damage healthcare facilities, rendering them inoperable at times when they are most needed. Flooding can inundate hospitals and clinics, leading to the loss of medical equipment, supplies, and patient records. Hurricanes and high winds can cause structural damage, requiring extensive repairs and rebuilding efforts.^[27]^ Healthcare facilities may face destruction or forced evacuations in areas prone to wildfires, further disrupting services. When healthcare facilities are damaged or destroyed, the continuity of HIV prevention and treatment services is severely compromised. Patients may lose access to lifesaving ART, leading to interruptions in treatment that can result in viral rebound and increased risk of transmission. Additionally, the loss of healthcare infrastructure can delay the diagnosis and management of new HIV infections, hindering efforts to control the epidemic.^[28]^

## 17. Disruption of supply chains

The impact of climate change on supply chains is another critical concern for HIV prevention and treatment. Extreme weather events can disrupt transportation networks, making it difficult to deliver medical supplies, including HIV medications, diagnostic tests, and preventive tools such as condoms and pre-exposure prophylaxis (PrEP).^[29]^ Flooded roads, damaged bridges, and other infrastructural challenges can delay or halt the shipment of essential supplies to affected areas. These disruptions can lead to shortages of HIV medications and other critical supplies, undermining the effectiveness of HIV programs. For individuals living with HIV, consistent access to ART is essential for maintaining viral suppression and preventing transmission. Supply chain disruptions can force patients to miss doses, potentially leading to drug resistance and treatment failure. Ensuring the resilience of supply chains is therefore vital for maintaining uninterrupted access to HIV care.^[30]^

## 18. Reallocation of resources

Climate-related emergencies often necessitate the reallocation of resources from long-term health programs to immediate disaster response efforts. During and after extreme weather events, governments and healthcare systems may prioritize emergency response activities, such as providing food, water, shelter, and medical care to affected populations. While these actions are essential for addressing immediate needs, they can divert funding and attention away from ongoing HIV prevention and treatment programs.^[31]^ The reallocation of resources can result in reduced funding for HIV programs, leading to cutbacks in services, outreach, and education efforts. This reduction in support can hamper the progress made in HIV prevention and treatment, increasing the risk of new infections and adverse health outcomes for people living with HIV. Balancing the demands of emergency response with the need for sustained investment in HIV programs is crucial for maintaining the momentum in the fight against HIV.^[5]^

## 19. Impact on healthcare workforce

Climate change also affects the healthcare workforce, which is essential for delivering HIV services. Extreme weather events can lead to displacement, injury, or loss of life among healthcare workers, reducing the available workforce in affected areas.^[25]^ Additionally, the stress and burnout associated with responding to climate-related emergencies can impact the mental health and well-being of healthcare providers, potentially leading to decreased productivity and increased turnover. A reduced and overburdened healthcare workforce can compromise the quality and availability of HIV services. Healthcare workers play a critical role in HIV prevention, testing, treatment, and support, and their well-being is essential for the effective functioning of healthcare systems. Supporting the resilience and mental health of healthcare workers is therefore an important component of maintaining robust HIV services in the face of climate change.^[18]^

## 20. Infrastructure resilience and adaptation

To address the challenges posed by climate change, it is essential to invest in resilient healthcare infrastructure that can withstand extreme weather events and ensure the continuity of HIV services. This includes building and retrofitting healthcare facilities to be more resistant to flooding, high winds, and other climate-related hazards. Incorporating renewable energy sources, such as solar power, can help ensure that healthcare facilities remain operational during power outages.^[18]^ Emergency preparedness plans are also critical for maintaining healthcare services during climate disruptions. These plans should include strategies for protecting and mobilizing medical supplies, ensuring communication with patients and healthcare workers, and establishing alternative care sites if primary facilities are damaged. Training healthcare workers in emergency response and resilience practices can further enhance the capacity of healthcare systems to manage climate-related challenges. Integrating climate resilience into HIV prevention strategies is a growing area of focus. The effectiveness of community-based programs that incorporate climate adaptation measures alongside HIV prevention efforts.^[32]^ These programs, which include education on sustainable practices and access to healthcare services, have shown promising results in enhancing community resilience and reducing HIV transmission rates. For instance, the “Sustainable Livelihoods Approach” implemented in several African countries has integrated HIV prevention with sustainable agriculture and economic empowerment, leading to improved health outcomes.^[32]^

## 21. Community-based resilience

Empowering communities to enhance their resilience to climate change can also support the sustainability of HIV services. Community-based approaches involve engaging local populations in disaster preparedness, health education, and resource mobilization.^[33]^ By building local capacity and fostering community ownership of health initiatives, these approaches can help ensure that HIV prevention and treatment efforts continue even in the face of climate-related disruptions. Community health workers and local organizations can play a vital role in maintaining HIV services during emergencies. They can provide outreach, education, and support to affected populations, helping to bridge gaps in healthcare access. Strengthening community-based networks and partnerships is essential for creating resilient health systems that can adapt to the impacts of climate change. Community engagement is essential for building resilience against climate change and HIV. Involving local communities in the design and implementation of health interventions can significantly enhance their effectiveness.^[34]^ Programs that empower communities to take ownership of their health, such as the “Community-Based Participatory Research” model, have been shown to improve health outcomes by fostering trust and increasing access to services. Additionally, community health worker programs have proven effective in providing education and resources related to both climate change and HIV prevention, thereby addressing the dual challenges simultaneously.^[34]^

## 22. Policy and advocacy

Policy and advocacy are crucial components of addressing the intersection of climate change and HIV prevention. Policymakers must recognize the interconnectedness of these issues and develop integrated strategies that address both climate resilience and HIV. This includes allocating sufficient funding for climate-adaptive healthcare infrastructure, supporting research on the impacts of climate change on HIV, and promoting policies that ensure the continuity of HIV services during emergencies. Global advocacy efforts are also needed to mobilize resources and political will to address^[28,29]^ the compounded challenges of climate change and HIV. International cooperation and partnerships can facilitate the sharing of best practices, knowledge, and resources to enhance the resilience of healthcare systems worldwide. Advocating for the inclusion of climate resilience in global health agendas can help ensure that the most vulnerable populations are protected. Empirical evidence underscores the need for comprehensive policy frameworks that integrate climate resilience into public health strategies. Cross-sector collaboration in developing policies that address the interconnectedness of climate change and health.^[8,18]^ Policymakers are encouraged to implement actions that promote sustainable healthcare practices, support community-driven initiatives, and invest in research on the impacts of climate change on HIV prevention. Furthermore, enhancing data collection and monitoring systems is vital for assessing the effectiveness of interventions and adapting strategies in response to emerging challenges.^[35]^

## 23. Integrating climate resilience into HIV prevention

The integration of climate resilience into HIV prevention is essential for developing sustainable health systems that can withstand the impacts of climate change. Given the interconnectedness of climate change and health, strategies that bolster resilience can help maintain the effectiveness of HIV prevention, testing, and treatment services even in the face of extreme weather events and other climate-related disruptions.^[15,30]^ A fundamental aspect of integrating climate resilience into HIV prevention is strengthening healthcare infrastructure to withstand extreme weather events. This includes both the physical resilience of healthcare facilities and the robustness of supply chains for medications and medical supplies. Investing in the construction and retrofitting of healthcare facilities to be more resistant to climate-related hazards is crucial. Ensuring that supply chains for HIV medications and other essential health supplies are resilient to disruptions is vital. Effective emergency preparedness and response plans are essential for maintaining HIV prevention and treatment services during and after climate-related emergencies. Conducting comprehensive risk assessments to identify vulnerabilities in healthcare infrastructure and services. Based on these assessments, developing detailed emergency response plans that outline specific actions to be taken before, during, and after extreme weather events.Providing training for healthcare workers and community health volunteers on emergency response and resilience practices. Involving communities in emergency preparedness efforts to enhance local capacity and resilience. Community members should be trained and equipped to support HIV services during emergencies, ensuring that they can help maintain continuity of care.^[36]^

Community-based approaches are crucial for integrating climate resilience into HIV prevention. These approaches focus on empowering local populations to adapt to climate changes and ensure the sustainability of HIV services. Encouraging communities to mobilize local resources and support networks to sustain HIV services during disruptions. Promoting health education and awareness programs that incorporate climate resilience messages.^[5,13]^ Supportive policies and advocacy efforts are essential for integrating climate resilience into HIV prevention on a larger scale. Policymakers and advocates must work to create an enabling environment that supports sustainable health systems. Developing policies that explicitly integrate climate resilience into health and HIV prevention programs. Engaging in global advocacy to mobilize resources and political will for addressing the intersection of climate change and HIV. Leveraging innovative approaches and technologies can significantly enhance the integration of climate resilience into HIV prevention. Utilizing mHealth technologies to deliver HIV services remotely, especially during climate-related disruptions. Implementing environmentally sustainable practices in healthcare facilities to reduce their carbon footprint and enhance resilience.^[34]^

## 24. Innovative approaches and sustainable solutions

To address the compounded challenges of climate change and HIV prevention, innovative approaches and sustainable solutions are imperative. Leveraging technology, embracing environmentally friendly practices, and fostering global cooperation can enhance resilience and ensure the continuity of HIV services.^[25]^

## 25. Mobile health (mHealth) technologies

Mobile health (mHealth) technologies are revolutionizing healthcare delivery, offering innovative solutions for HIV prevention, especially in regions affected by climate change. These technologies can provide remote access to health services, ensuring continuity of care during and after extreme weather events.^[16]^Telemedicine allows healthcare providers to conduct remote consultations and follow-ups with patients. This is particularly valuable during climate-related disruptions when physical access to healthcare facilities may be limited. Telemedicine can help maintain ART adherence, provide counseling, and manage other health needs of people living with HIV. Mobile applications can support patients in adhering to their medication regimens by providing reminders and educational content. Apps can also facilitate communication between patients and healthcare providers, enabling timely interventions if issues arise.SMS (Short Message Service) systems can disseminate important information and alerts to patients and communities. These systems can be used to send reminders for medication, appointments, and to provide health education. During emergencies, SMS can be a critical tool for disseminating information about available services and resources.^[34]^

## 26. Green healthcare initiatives

Adopting environmentally sustainable practices in healthcare facilities is essential for reducing their carbon footprint and enhancing their resilience to climate change. Green healthcare initiatives not only contribute to environmental sustainability but also ensure the long-term viability of health services. Implementing renewable energy solutions, such as solar or wind power, can ensure that healthcare facilities remain operational during power outages caused by extreme weather events. Renewable energy sources also reduce the dependency on fossil fuels, contributing to overall climate change mitigation efforts.^[25]^Utilizing energy-efficient lighting, heating, cooling, and medical equipment can significantly reduce energy consumption in healthcare facilities. Energy efficiency measures can lower operational costs and make facilities more sustainable. Constructing and retrofitting healthcare facilities with sustainable materials and practices can enhance their resilience to climate-related hazards. Green building designs that incorporate natural lighting, ventilation, and water conservation measures can create healthier and more sustainable environments for both patients and healthcare workers.^[16,20]^

## 27. Integrated health and environmental programs

Combining health and environmental programs can create synergies that enhance both climate resilience and HIV prevention efforts. Integrated approaches can address the root causes of vulnerability and promote sustainable development. Programs that promote sustainable agriculture and improve food security can indirectly support HIV prevention. Adequate nutrition is essential for the health of people living with HIV, and food security can reduce the need for high-risk behaviors associated with food scarcity.^[16]^ Community gardens, small-scale farming, and nutritional education programs can empower communities to become more self-sufficient and resilient. Improving access to clean water, sanitation, and hygiene can prevent the spread of infections and enhance overall health outcomes. WASH initiatives can reduce the burden on healthcare systems and create healthier living environments, which are critical for effective HIV prevention and care.^[34,36]^

## 28. Climate-resilient healthcare systems

Building climate-resilient healthcare systems involves creating infrastructures and practices that can withstand and adapt to the impacts of climate change. This requires a multifaceted approach that integrates emergency preparedness, flexible service delivery, and robust healthcare policies. Developing comprehensive emergency preparedness plans is crucial for ensuring the continuity of HIV services during climate-related disruptions.^[1]^ These plans should include strategies for protecting healthcare infrastructure, securing medical supplies, and maintaining communication with patients and healthcare workers. Implementing flexible service delivery models, such as mobile clinics and community health worker programs, can ensure that HIV services reach vulnerable populations even when traditional healthcare facilities are compromised.^[31]^ These models can be quickly adapted to changing circumstances and provide critical care in hard-to-reach areas. Advocating for policies that support climate resilience in healthcare systems is essential for sustainable HIV prevention. This includes securing funding for resilient infrastructure, promoting cross-sector collaboration, and integrating climate considerations into health policies. Policymakers must prioritize long-term investments in healthcare resilience to protect against future climate impacts.^[18,32]^

## 29. Global cooperation and knowledge sharing

Global cooperation and knowledge sharing are vital for addressing the intersection of climate change and HIV prevention. Collaborative efforts can facilitate the exchange of best practices, resources, and innovations, enhancing the resilience of healthcare systems worldwide.^[4,9]^Forming international partnerships and coalitions can help mobilize resources and expertise to support climate-resilient HIV prevention efforts. Global health organizations, governments, and NGOs can work together to develop and implement effective strategies. Supporting research on the impacts of climate change on HIV and developing innovative solutions is crucial for advancing knowledge and practice. Research can identify effective interventions, inform policy decisions, and guide the development of new technologies and approaches.^[16]^Creating platforms for knowledge sharing and capacity building can enable healthcare providers, policymakers, and communities to learn from each other’s experiences. Conferences, workshops, and online forums can facilitate the dissemination of information and foster collaborative problem-solving.^[34–36]^

## 30. Recommendations

To effectively address the challenges posed by climate change on HIV prevention, the following recommendations are proposed for various stakeholders, including policymakers, healthcare providers, researchers, and community organizations:

## 31. Integrate climate change considerations into health policies

Ensure that national and local health policies explicitly incorporate climate change impacts, addressing how environmental changes can affect health outcomes, particularly for vulnerable populations at risk for HIV. Foster collaboration between health, environmental, and disaster management sectors to create comprehensive strategies that address the interconnectedness of climate change and health.

## 32. Enhance healthcare infrastructure resilience

Allocate resources to build and retrofit healthcare facilities that are resilient to climate change, using environmentally sustainable materials and designs to withstand extreme weather events. Develop and implement robust emergency preparedness plans that prioritize the continuity of HIV services during climate-related disasters.

## 33. Strengthen HIV service delivery in vulnerable communities

Implement community-centered HIV prevention programs that consider local environmental challenges and incorporate climate adaptation strategies. Ensure that marginalized populations have access to comprehensive HIV services, including testing, treatment, and education, especially during climate-related emergencies.

## 34. Promote education and awareness

Develop and disseminate educational materials that raise awareness of the impacts of climate change on health and HIV prevention, targeting both healthcare providers and the general public. Provide training for healthcare professionals on the intersections of climate change and HIV prevention, equipping them to address the unique challenges faced by their communities.

## 35. Foster research and innovation

Encourage research that explores the impacts of climate change on HIV prevention and treatment, including studies on effective interventions and community-based strategies. Invest in the development and deployment of innovative technologies (e.g., mHealth solutions) that can improve access to HIV services and facilitate data collection during climate emergencies.

## 36. Build community capacity and resilience

Engage communities in the design and implementation of health programs that address both HIV prevention and climate adaptation, fostering local ownership and capacity building. Promote sustainable agricultural and economic practices that enhance food security and reduce vulnerability to climate change, thereby indirectly supporting HIV prevention efforts.

## 37. Advocate for inclusive policies

Implement policies that specifically address social determinants of health and health disparities exacerbated by climate change, ensuring equitable access to healthcare for all populations. Recognize and address the unique vulnerabilities faced by women and girls in the context of climate change and HIV, promoting gender-sensitive policies and interventions.

## 38. Monitor and evaluate interventions

Establish systems for collecting and analyzing data on the impacts of climate change on HIV health outcomes, allowing for evidence-based decision-making and policy refinement. Regularly evaluate the effectiveness of climate-resilient HIV prevention strategies and programs, using findings to inform future initiatives and improve service delivery.

## 39. Conclusion

The intersection of climate change and HIV prevention presents a complex challenge that requires immediate and coordinated action. As climate change continues to exacerbate health vulnerabilities, it is crucial to recognize its impact on HIV prevention, testing, and treatment services. Integrating climate resilience into HIV prevention efforts is not only a matter of protecting health outcomes but also of promoting social equity and safeguarding the rights of marginalized populations. By investing in resilient healthcare infrastructure, strengthening supply chains, enhancing emergency preparedness, and fostering community engagement, we can create healthcare systems that are better equipped to withstand the impacts of climate change. Furthermore, adopting green healthcare practices and leveraging technology will contribute to both health and environmental sustainability, ensuring that HIV services remain accessible and effective. Policy recommendations that prioritize integrated approaches, global cooperation, and inclusive practices are essential for creating a supportive environment for climate-resilient HIV prevention. By mobilizing resources, engaging communities, and advocating for equitable policies, we can work towards a future where health systems are not only resilient to climate change but also effective in combating HIV.

## Author contributions

**Conceptualization:** Emmanuel Ifeanyi Obeagu.

**Methodology:** Emmanuel Ifeanyi Obeagu, Getrude Uzoma Obeagu.

**Resources:** Emmanuel Ifeanyi Obeagu.

**Supervision:** Emmanuel Ifeanyi Obeagu.

**Validation:** Emmanuel Ifeanyi Obeagu, Isaac Isiko, Getrude Uzoma Obeagu.

**Visualization:** Emmanuel Ifeanyi Obeagu, Isaac Isiko, Getrude Uzoma Obeagu.

**Writing – original draft:** Emmanuel Ifeanyi Obeagu, Isaac Isiko, Getrude Uzoma Obeagu.

**Writing – review & editing:** Emmanuel Ifeanyi Obeagu, Isaac Isiko, Getrude Uzoma Obeagu.
